# Does More Respect from Leaders Postpone the Desire to Retire? Understanding the Mechanisms of Retirement Decision-Making

**DOI:** 10.3389/fpsyg.2017.01400

**Published:** 2017-08-23

**Authors:** Anne M. Wöhrmann, Ulrike Fasbender, Jürgen Deller

**Affiliations:** ^1^Federal Institute for Occupational Safety and Health (BAuA) Dortmund, Germany; ^2^Work and Organizational Psychology, Justus-Liebig-University Giessen Giessen, Germany; ^3^Institute for Strategic HR Management Research and Development, Leuphana University Lüneburg, Germany

**Keywords:** desired retirement age, job satisfaction, occupational self-efficacy, respectful leadership, subjective health, work-to-private life conflict

## Abstract

The demographic trends (i.e., low birth rates and increasing longevity) pose challenges with regard to the increase of the average employee age along with a lack of skilled personnel on the labor market. Society, organizations, and individuals are confronted with the question on how to prolong working lives in the future. Based on socioemotional selectivity theory, the purpose of this study was to investigate the relationship between respectful leadership and older workers’ desired retirement age. In particular, we took a closer look at job satisfaction, subjective health, and work-to-private life conflict as underlying mechanisms. Further, we tested for the moderating role of occupational self-efficacy as an auxiliary condition for the assumed relationships of respectful leadership. We tested our hypothesized model using data from 1,130 blue- and white-collar workers aged 45–65 years. The results of structural equation modeling indicated that respectful leadership was positively related to older workers’ desired retirement age and that this relationship was mediated by subjective health and work-to-private life conflict but not by job satisfaction. The findings add to the literature on resources in retirement decision-making; notably, they highlight the importance of leadership behavior for older workers’ motivation and socioemotional needs.

## Introduction

Demographic trends such as low birth rates and increasing longevity pose challenges with regard to the increase of the average employee age along with a lack of skilled personnel on the labor market. Society, organizations, and individuals are confronted with the question of how to develop conditions to prolong working lives in the future. As jobs often include demands that have negative impacts on older workers’ health or conflict with their changing abilities and non-work obligations, many of them aspire to retire early (Topa et al., 2009). Scholars have emphasized that pressuring people to continue working for financial reasons only may result in negative consequences such as lower levels of well-being (Frins et al., 2016). Maintaining older people’s ability and motivation to work is therefore an essential condition for the extension of working lives.

Many theories (e.g., rational choice theory, role theory, theory of planned behavior, and expectancy theory) refer to retirement decision-making as a highly rational process in a way that long-term goals are maximized, while costs are minimized (Jex and Grosch, 2012; Wang and Shi, 2014). However, those theories often neglect that as people age their motivation changes. According to socioemotional selectivity theory (SST; Carstensen, 1992, 2006) with increasing age, people perceive time as more limited and therefore, place greater importance on short-term goals from which they derive emotional meaning. Following this, positive interpersonal relationships at work are meaningful in allowing older workers to maximize their emotional and social gains while limiting emotional and social risks at work. The desire to retire as opposed to remain in work is therefore evaluated against its potential for offering a positive balance of socioemotional gains and risks.

This and other changes in motivations as people age call for the need to develop and implement age sensitive human resource practices (e.g., Kooij et al., 2008; Wöhrmann et al., 2016). Older workers tend to favor working conditions that allow them to experience meaningfulness and recognition at work (Zacher, 2015; Hertel and Zacher, 2016). A leader who shows recognition and respect toward his or her subordinates may enhance older workers’ social and emotional gains in the workplace. In this study, we therefore take a closer look at respectful leadership as a potential age sensitive leadership behavior. Drawing on SST (Carstensen, 1992, 2006), we argue that respectful leadership could be a source to meet socioemotional needs of workers and thus act as a contextual resource that affects older workers’ desired retirement age by enhancing subjective health, job satisfaction, and lowering work-to-private life conflict.

**FIGURE 1:**
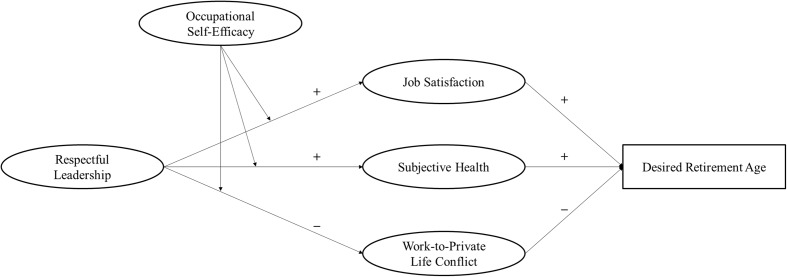
Conceptual model indicating the hypothesized relationships between respectful leadership, job satisfaction, subjective health, work-to-private life conflict, occupational self-efficacy, and desired retirement age.

In addition, we investigate the moderating role of occupational self-efficacy to understand whether this personal resource can support the goal-directed use, acquisition, and maintenance of other personal resources. As a result, our findings contribute to the understanding of the interplay between contextual and personal resources in the retirement decision-making process. Regarding organizational practice, we contribute to the development of human resource management strategies that support the extension of working lives. The conceptual model is presented in **Figure 1**.

## Respectful Leadership and Older Workers’ Desired Retirement Age

Retirement decision-making is a process, in which the desired retirement age is an expression of the intention or the preference to retire at a certain age in the future. The desired retirement age as an early step in the retirement decision-making process has been described as the most proximal predictor of the actual act of retirement (Beehr, 1986), which leads to the search for predictors of desired retirement age.

In their resource-based dynamic model for retirement adjustment, Wang et al. (2011) propose that retirement-related outcomes are the result of access to and interplay between different resources. Resources – as supportive factors that people value – can be categorized into contextual and personal resources (ten Brummelhuis and Bakker, 2012). Drawing on SST, appreciation and respect in the workplace represent important contextual resources in the retirement decision-making process, which help to meet socioemotional needs of older workers. This is supported by research showing that being respected and recognized at work is a relatively important aspect to continue working (e.g., Armstrong-Stassen, 2008; Pundt et al., 2015; Zhan et al., 2015; Fasbender et al., 2016). Thus, experienced respectful treatment by the leader may act as a contextual resource in the retirement decision-making process.

The construct of respectful leadership was introduced by van Quaquebeke and Eckloff (2010) based on the definition of respect as “a person’s attitude towards other people, in whom he/she sees a reason that, in itself, justifies a degree of attention and a type of behavior that in return engenders in the target a feeling of being appreciated in importance and worth as a person” (p. 344). Respectful leadership reflects leader behaviors and attitudes that give employees the feeling of being respected. Examples of respectful leadership behaviors include the recognition of the employees and their work, interest in their opinions, polite and fair treatment of the employees, open and honest interaction, support, as well as the recognition of the individual situation of the employees.

That respectful leadership may play an important role for retirement decisions is supported by leader–member exchange theory (LMX; Graen et al., 1982) highlighting the quality of the dyadic relationship between leader and follower. According to LMX, the relationship quality develops through the exchange of resources of leader and subordinate with a high-quality relationship being characterized by mutual trust and respect (Bauer and Green, 1996). Thus, professional respect as one dimension of LMX constitutes a central element in the quality of the relationship between leader and follower (Liden and Maslyn, 1998). Research suggests LMX to be related to withdrawal intentions (e.g., Gerstner and Day, 1997). Therefore, it is not unlikely that respectful leadership (as one aspect of LMX) may influence older workers’ retirement decisions.

### Respectful Leadership and Its Relationship to Job Satisfaction, Subjective Health, and Work-to-Private Life Conflict

Contextual resources can facilitate the acquisition of personal resources. Having a respectful leader may trigger the gain of other resources that could be relevant for older workers. Against the framework of SST, that due to limited time horizons older people increasingly value positive emotional experiences and are less likely to accept emotional burdens at work, thus, job satisfaction can constitute a relevant resource. Another important resource that becomes more evident with age is health. Moreover, as people age they increasingly value emotionally meaningful relationships and may therefore be inclined to spend more quality time with significant others. Therefore, in the following the potential role of respectful leadership for job satisfaction, health, and work-to-private life conflict is outlined.

Being treated with respect may enhance social and emotional gains and consequently contribute to older workers’ job satisfaction. Thus, the experienced behavior *per se* that creates a feeling of being respected for one’s work may lead to a gain in resources, for example related to the individuals’ mood, by creating positive feelings toward the job. This assumption is supported by the importance of affective events at work (Weiss and Cropanzano, 1996). Emotional reactions to events in the work environment can affect individuals’ attitudes and behaviors. Respectful leadership behavior may provoke such affective events that are followed by a positive cognitive appraisal by the subordinate resulting in a positive affective reaction. Also, respectful behavior of the leader may lead to favorable working conditions which are related to favorable workplace outcomes such as job satisfaction as has been shown by previous research (van Quaquebeke and Eckloff, 2010). We therefore expect that respectful leadership is positively related to older workers’ job satisfaction.

Further, the contextual resource of feeling respected by the leader can have beneficial effects on the personal resource employee health by reducing strain. Respectful leader behavior includes trust and protection which can help preventing stress reactions linked to employee health. For example, effectively using a person’s potential and best abilities instead of focusing on declining abilities is likely to reduce strain on the individual. The positive relationship of respectful leadership and health is supported by a recent study by Montano (2016), who found respectful leader behavior to be linked to better work designs, and hence to employees’ self-assessed general health. Respectful leader behavior has also been suggested to promote employees’ health in jobs with a poor work design (Winkler et al., 2014). It is therefore plausible to assume that respectful leadership is positively related to older workers’ subjective health.

Furthermore, spending valuable time with family and friends can provide an important personal resource for older workers. Also, older workers increasingly need to meet care demands – for their grandchildren, children, parents, or other relatives (Szinovacz and Davey, 2005). Conflicts between work and private life result from scarce time resources, strain (e.g., fatigue), or contradictory expectations regarding behavior related to different roles (Greenhaus and Beutell, 1985). Bearing in mind that individuals have other obligations and interests in their private life apart from their family, the construct of work–family conflict can be expanded to work-to-private life conflict to capture these as well.

Even though respectful leadership is unlikely to affect older workers’ private commitments, a leader who treats the subordinates with respect can be beneficial for reducing their job strain. As noted by Bakker and Demerouti (2007): “leaders’ appreciation and support puts demands in another perspective” (Bakker and Demerouti, 2007, p. 315) not least because a respectful leader may help enhance social and emotional gains in the workplace. A respectful leader, who is interested in the employees and understands their demands, may help minimize conflicts between work and private life by giving the employees working time control. Also, the feeling of being treated respectfully may result in less negative spillovers from work to private life, for example in terms that the employees do not feel as stressed or fatigued because they derive positive feelings from feeling being trusted, appreciated, and fairly treated.

Based on the considerations on resource gains and maintenance through respectful leadership, we propose the following hypothesis:

Hypothesis 1: Respectful leadership is positively related to (a) job satisfaction and (b) subjective health, but negatively related to (c) work-to-private life conflict.

### Job Satisfaction, Subjective Health, Work-to-Private Life Conflict, and Their Relationship to Desired Retirement Age

In the face of loss or threat of personal or contextual resources individuals reduce their investment or invest in behaviors that are more strategic in their use of resources (Halbesleben et al., 2014). Thus, in the case of older workers this investment decision may impact their planned retirement age. A satisfying job provides a valuable resource (Wright and Bonett, 2007), which in turn can facilitate the creation and preservation of other personal resources. On the one hand, continued employment can help to preserve these personal resources. On the other hand, older workers who are not satisfied with their job may experience loss of these personal resources and thus intent to change the situation by intending to retire early. Furthermore, according to SST older people are less likely to accept emotional burdens at work. Therefore, it is likely that older workers’ desired retirement age depends on their job satisfaction. Previous research supports this contention as job satisfaction has been found to be associated with the ability to remain working until the retirement age (Maurits et al., 2015).

Moreover, subjective health has been found to be an important factor for remaining in the workforce (e.g., Fasbender et al., 2014; Zaniboni, 2015). Subjective health (also self-reported health) refers to one’s overall evaluation of physical well-being, which is strongly related to physical, functional, and mental health (Pinquart, 2001). Health is a personal resource *per se* and if employees experience their health to worsen they feel a resource loss. If they feel this resource loss to be due to their work, early retirement intentions become likely. For example, losses in subjective health positively predict limited future time perspective (Kooij and van de Voorde, 2011) and work engagement (Kooij et al., 2013). Also, as good health is a necessary condition to continue working, it is likely that subjective health is positively related to older workers’ desired retirement age.

Balancing work with one’s private life is a challenge every employee has to face. Several resources, such as mood, time, and the social network, may be threatened if older workers experience work-to-private life conflict. Depending on how much the affected resources are valued by the employee, this may result in early retirement plans. According to SST as individuals age and perceive time as more limited, they place greater importance on emotionally meaningful relationships, and therefore family often becomes more important relative to work. This is in line with assumptions of Baltes and Baltes (1990) model of successful aging drawing on regulation processes of selection, optimization, compensation (SOC) with regard to ones goals: Due to limited resources older workers have to carefully select their goals and roles to age successfully. Thus, work-to-private life conflict is likely to reduce older workers’ desired retirement age. A recent longitudinal study by Nohe and Sonntag (2014) reported that work–family conflicts may increase turnover intentions. With regard to older workers, research suggests that care responsibilities lead to early retirement, especially among women (Lumsdaine and Vermeer, 2014). For older workers (early) retirement can be an option to lower work demands, which helps to deal with arising private commitments and thus, alleviate the anticipated role pressure between work and private life. It is therefore likely that work-to-private life conflict is negatively related to desired retirement age.

To summarize, our second hypothesis reads:

Hypothesis 2: (a) Job satisfaction and (b) subjective health are positively related to desired retirement age, but (c) work-to-private life conflict is negatively related to desired retirement age.

### Respectful Leadership and Its Indirect Relationship to Desired Retirement Age

Having introduced job satisfaction, subjective health, and work-to-private life conflict as underlying mechanisms we now draw the link between respectful leadership and desired retirement age. In particular, we argue that there is a positive but indirect relationship between respectful leadership and desired retirement age, which is expected to be explained by resource gains or losses, respectively, due to increased or reduced levels of job satisfaction, subjective health, and work-to-private life conflict. This is emphasized by the notion that every individual is “a proactive agent for his or her career development” (Fasbender and Deller, 2017, p. 729), indicating that older workers do not directly react to certain external factors (e.g., respectful leadership). Rather, they respond through the internal process of evaluating their level of resources such as their job satisfaction, health, and role conflicts that materialize as a consequence of simultaneously occurring work demands and private commitments. This in turn may influence older workers’ desired retirement age. Among others, these assumptions are supported by research on LMX that shows that the effect of quality of the relationship between leader and follower on turnover intentions is mediated by job satisfaction (Han and Jekel, 2011). Therefore, it is plausible to assume that a positive relationship between respectful leadership and desired retirement age can be explained by higher levels of job satisfaction and subjective health but lower levels of work-to-private life conflict; hence, our third hypothesis reads:

Hypothesis 3: There is a positive indirect relationship between respectful leadership and desired retirement age via (a) job satisfaction, (b) subjective health, and (c) work-to-private life conflict.

### The Moderating Role of Occupational Self-Efficacy

Occupational Self-Efficacy has been derived from the general construct of self-efficacy reflecting the beliefs individuals hold about their capabilities to manage situations and produce designated levels of performance to reach their goals with regard to their job (Rigotti et al., 2008). It has been found to be associated with several outcomes among which are job satisfaction (Schyns and Collani, 2002; Rigotti et al., 2008) and burnout (Guglielmi et al., 2012). Despite much research on self-efficacy, to date, little is known about the role that occupational self-efficacy plays for older workers (Paggi and Jopp, 2015).

We argue that occupational self-efficacy can support the goal-directed use, acquisition, and maintenance of other personal resources, which helps explain why some individuals are better or more effective, respectively, at building and using resources. During their working life, people gain expertise and trust in their own abilities which should contribute to strong occupational self-efficacy beliefs. However, due to differences in the work design, possibilities, and abilities, this is not true for every individual. Older workers with high occupational self-efficacy beliefs are convinced that they have the ability to perform well in their work tasks. Higher levels of occupational self-efficacy lead people taking an active role in interpreting external factors (Lent et al., 1994; Fasbender and Deller, 2017). Older workers with high occupational self-efficacy beliefs possess a resource that helps them use respectful leadership as a contextual resource to maintain and gain other resources, in particular job satisfaction, health, and work–life balance. Therefore, older workers with high occupational self-efficacy beliefs are more likely to benefit from respectful leadership compared to older workers with low occupational self-efficacy beliefs; finally, our fourth hypothesis reads:

Hypothesis 4: Occupational self-efficacy moderates the relationships of respectful leadership with (a) job satisfaction, (b) subjective health, and (c) work-to-private life conflict in a way that the relationships are stronger when occupational self-efficacy is high (vs. low).

## Materials and Methods

### Sample and Procedure

As part of a larger project on aging and work, data were collected from a large logistics company with several sites in Germany. All of the employees aged 45–65 years in the company were invited to participate in the study. Half of them (50%) voluntarily agreed to participate, resulting in a sample of 1,130 employees. Participants from operative departments, such as incoming returns, commissioning, or product testing, without a computer work-space (i.e., blue-collar workers) completed paper–pencil questionnaires (*n* = 830; 73.5%), and participants with a computer work-space from administrative departments (i.e., white-collar workers) completed online questionnaires (*n* = 300; 26.5%). Of the participants, the majority (68.8%) were female. Their age ranged from 45 to 65 years, with a mean age of 51.43 years (*SD* = 4.29). About a fifth of participants (22.0%) had obtained higher education entrance qualifications and/or graduated from college or university.

### Measures

#### Respectful Leadership

Respectful leadership was measured with the Respectful Leadership Scale developed by van Quaquebeke and Eckloff (2010). The scale contains 12 items, an example item is “My leader takes me and my work seriously.” The participants responded on a five-point Likert scale from 1 (*does not apply at all*) to 5 (*applies completely*). In the current study, the scale showed a high internal consistency (Cronbach’s α = 0.94). Furthermore, results of a confirmatory factor analysis supported the construct validity of the one-factor solution [χ^2^(54) = 376.90, *p* < 0.01, comparative fix index (CFI) = 0.94, root mean square error of approximation (RMSEA) = 0.07, standardized root mean square residual (SRMR) = 0.03].

#### Job Satisfaction

Job satisfaction was measured with the general work satisfaction subscale of the German version of the Job Diagnostic Survey (Schmidt et al., 1985). This subscale consists of five items. An example item is “Generally speaking, I am very satisfied with my work.” The participants responded on a five-point Likert scale from 1 (*does not apply at all*) to 5 (*applies completely*). In this study, the subscale showed an acceptable internal consistency (Cronbach’s α = 0.73) and overall an acceptable model fit in the confirmatory factor analysis (χ^2^(5) = 73.99, *p* < 0.01, CFI = 0.94, RMSEA = 0.11, SRMR = 0.05).

#### Subjective Health

Subjective health was measured with three items regarding self-rated general health, health in comparison with others, and today’s health. The response format was a five-point Likert scale from 1 (*very bad/much worse*) to 5 (*very good*/*much better*). The measure showed a good internal consistency (Cronbach’s α = 0.83) in this study.

#### Work-to-Private Life Conflict

Work-to-private life conflict was measured with the five items Work–Family Conflict Scale developed by Netemeyer et al. (1996). As not every person has a family we changed “family” to “private.” An example item is “The demands of my work interfere with my home and private life.” The participants responded on a five-point Likert scale from 1 (*does not apply at all*) to 5 (*applies completely*). Internal consistency was high (Cronbach’s α = 0.91) and construct validity was supported by the results of a confirmatory factor analysis (χ^2^(5) = 33.46, *p* < 0.01, CFI = 0.99, RMSEA = 0.07, SRMR = 0.02) in this study.

#### Occupational Self-Efficacy

The Occupational Self-Efficacy Short Scale in German (Rigotti et al., 2008) was applied to measure occupational self-efficacy. The scale consisted of six items. An example item is “Whatever comes my way in my job, I can usually handle it.” The participants responded on a five-point Likert scale from 1 (*does not apply at all*) to 5 (*applies completely*). The short scale showed a good internal consistency (Cronbach’s α = 0.85) and an acceptable model fit in a confirmatory factor analysis (χ^2^(9) = 90.45, *p* < 0.01, CFI = 0.93, RMSEA = 0.09, SRMR = 0.04).

#### Desired Retirement Age

Participants were asked to state their desired retirement age in years, which is common practice in research on retirement decision-making (e.g., HILDA Survey; Zaniboni, 2015). While the desired retirement age ranged from 48 to 80 years (*M* = 60.78, *SD* = 2.73), it was noticeable that more than half of the participants (55.1%) stated 60 years to be their desired retirement age, indicating limited variance.

#### Control Variables

As the retirement decision-making process may be affected by individuals’ age, gender, and education (e.g., Zaniboni, 2015; Fasbender et al., 2016), these variables were included in the analyses. Similarly, because previous research on blue-collar workers (Szubert and Sobala, 2005) indicated that certain work conditions (e.g., heavy lifting at work) were related to early workforce exit, we also controlled for participants’ function (blue- vs. white-collar workers) in the company.

## Results

### Preliminary Analysis

Means, standard deviations, and correlations of the study variables are presented in **Table 1**. Among the control variables, participants’ age and gender (0 = *female*, 1 = *male*) were positively correlated with desired retirement age but not education and function. With regard to the predictor variables, respectful leadership, job satisfaction, subjective health, and occupational self-efficacy were positively correlated with desired retirement age, whereas work-to-private life conflict was negatively correlated with desired retirement age.

**Table 1:** Means, standard deviations, and correlations of study variables (*N* = 1,046–1,130).

Variable	*M*	*SD*	1	2	3	4	5	6	7	8	9
(1) Age	51.43	4.29	–								
(2) Gender (0 = female, 1 = male)	0.31	0.46	-0.03	–							
(3) Education (0 = low, 1 = high)	0.22	0.41	0.04	0.14^∗∗^	–						
(4) Function (0 = blue, 1 = white)	0.27	0.44	-0.07^∗^	0.11^∗∗^	0.34^∗∗^	–					
(5) Respectful leadership	3.93	0.88	-0.01	-0.09^∗∗^	-0.04	0.09^∗∗^	–				
(6) Job satisfaction	3.63	0.77	0.03	0.04	-0.03	0.16^∗∗^	0.45^∗∗^	–			
(7) Subjective health	3.56	0.85	-0.02	0.05	0.05	0.09^∗∗^	0.25^∗∗^	0.37^∗∗^	–		
(8) Work-to-private life conflict	3.29	1.15	-0.03	-0.08^∗∗^	0.01	-0.11^∗∗^	-0.19^∗∗^	-0.34^∗∗^	-0.36^∗∗^	–	
(9) Occupational self-efficacy	3.88	0.74	0.01	0.07^∗^	-0.00	0.08^∗∗^	0.38^∗∗^	0.38^∗∗^	0.35^∗∗^	-0.20^∗∗^	–
(10) Desired retirement age	60.78	2.73	0.17^∗∗^	0.12^∗∗^	0.06	0.00	0.11^∗∗^	0.17^∗∗^	0.25^∗∗^	-0.17^∗∗^	0.12^∗∗^


*^∗^*p* < 0.05; ^∗∗^*p* < 0.01.*

### Testing Direct and Indirect Main Effects

Structural equation modeling (SEM) was used to investigate the hypothesized relationships between respectful leadership, work-to-private life conflict, occupational self-efficacy, job satisfaction, subjective health, and desired retirement age, using Mplus 7.31 (Muthén and Muthén, 2015). Within the structural part of SEM, we used maximum likelihood (ML) estimation with bootstrapping (10,000 draws) to account for deviations from normality (Preacher and Hayes, 2008). To assess whether the hypothesized model fits the data, we have used different goodness-of-fit indices. In addition to the traditional chi-square value as an index of absolute (lack of) fit, which, however, is sensitive to sample size (Bentler and Bonnet, 1980), we also report the CFI (Bentler, 1990), the SRMR, and the RMSEA (Steiger, 1990). As none of these goodness-of-fit indices is perfectly informative on its own, combining them is instructive to evaluate the model fit. To gauge the model fit in the present study, we have included participants’ age, gender, and function as control variables in the SEM. Job satisfaction, subjective health, work-to-private life conflict, and desired retirement age were regressed on these variables. Participants’ education has not been included as control variable because the preliminary analysis indicated that it was not correlated to any of the outcome variables. Overall, our hypothesized model including control variables showed a fairly good model fit (χ^2^(569) = 2454.02, *p* < 0.01, CFI = 0.91, RMSEA = 0.06, SRMR = 0.09).

**FIGURE 2:**
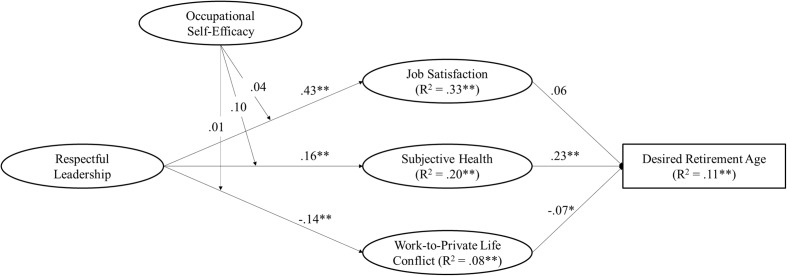
Structural equation model showing relationships between respectful leadership, work-to-private life conflict, occupational self-efficacy, job satisfaction, subjective health, and desired retirement age with standardized coefficients (*N* = 1,088). For the stake of clarity, only structural relationships are shown. ^∗^*p* < 0.05, ^∗∗^*p* < 0.01.

In the following, we refer to the standardized estimates of our hypothesized model as can be seen in **Figure 2**. Together, the predictor variables explained substantial variance in older workers’ desired retirement age. Among the control variables, structural coefficients suggested that age (β = 0.17, *p* < 0.01) and gender (β = 0.10, *p* < 0.01) were positively related to desired retirement age, while function did not significantly predict older workers’ desired retirement age. Furthermore, gender (β = -0.07, *p* < 0.05) and participants’ function (i.e., white collar) (β = -0.10, *p* < 0.01) were negatively associated with work-to-private life conflict, whereas age did not significantly predict work-to-private life conflict. Also, none of the control variables significantly predicted job satisfaction or subjective health.

Hypotheses 1–3 addressed the direct and indirect relationships between respectful leadership and desired retirement age. The structural coefficients suggested that respectful leadership was positively related to job satisfaction (β = 0.43, *p* < 0.01) and subjective health (β = 0.16, *p* < 0.01), but negatively related to work-to-private life conflict (β = -0.14, *p* < 0.01). Together, these results support Hypotheses 1a–c and indicate that older workers, who experience higher levels of respectful leadership, are more likely to be satisfied with their job, show higher levels of subjective health, and report lower levels of work-to-private life conflict compared to older workers, who experience lower levels of respectful leadership. In turn, the structural coefficients suggested that job satisfaction (β = 0.06, n.s.) was not related to desired retirement age. Thus, Hypothesis 2a was not supported. Subjective health (β = 0.23, *p* < 0.01) was positively related to desired retirement age and work-to-private life conflict (β = -0.07, *p* < 0.05) was negatively related to desired retirement age, supporting Hypotheses 2b and 2c. This indicates that higher levels of subjective health are likely to increase older workers’ desired retirement age, whereas higher levels of work-to-private life conflict decrease older workers’ desired retirement age. With regard to the relationship between respectful leadership and desired retirement age, we quantified the indirect effects with the product of coefficients method (Preacher, 2015). We found positive indirect effects of respectful leadership via subjective health (β = 0.04, *p* < 0.01) and via work-to-private life conflict (β = 0.01, *p* < 0.05) but not via job satisfaction (β = 0.03, n.s.), on desired retirement age. **Table 2** indicates the indirect effects of respectful leadership on desired retirement age with bootstrapped confidence intervals.

**Table 2:** Indirect effects of respectful leadership on desired retirement age with bootstrapped confidence intervals.

	Coefficient	SE	CI LL	CI UL
Job satisfaction	0.12 (0.03)	0.08 (0.02)	-0.02	0.28
Subjective health	0.17 (0.04)	0.05 (0.01)	0.10	0.25
Work-to-private life conflict	0.04 (0.01)	0.03 (0.01)	0.00	0.11


**N* = 1,088. CI = 95% confidence interval, LL = lower level, UL = upper level. Standardized coefficients in brackets.*

To test whether these variables partially or fully explain the positive relationship between respectful leadership and desired retirement age, we ran an alternative model, including a direct path from respectful leadership to desired retirement age. The direct path was, however, not significant (β = 0.02, n.s.), while the indirect relationships remained fairly stable. Furthermore, we found no significant differences between partial and full mediation models using chi-square difference testing (Δχ^2^(1) = 0.19); thus, we kept the more parsimonious, fully mediated model. Together, these results support Hypothesis 3b and 3c, and indicate that there is a positive relationship between respectful leadership and older workers’ desired retirement age, which can be explained by subjective health and work-to-private life conflict.

### Testing Moderation Effects

Hypothesis 4 addressed the moderating role of occupational self-efficacy. To test this hypothesis, we regressed job satisfaction, subjective health, and work-to-private life conflict on occupational self-efficacy in the SEM. The estimated coefficients showed that occupational self-efficacy was positively related to job satisfaction (β = 0.37, *p* < 0.01) and subjective health (β = 0.40, *p* < 0.01) but negatively related to work-to-private life conflict (β = -0.21, *p* < 0.01). To test the interaction we included a single indicator in the SEM that was computed as the product of the mean centered scale scores of respectful leadership and self-efficacy following the approach from Jöreskog and Yang (1996). Job satisfaction, subjective health, and work-to-private life conflict were then regressed on this indicator in the SEM. However, occupational self-efficacy did not significantly moderate the relationships between respectful leadership and job satisfaction, subjective health, and work-to-private life conflict, thus, Hypothesis 4 was not supported.

## Discussion

As demographic trends such as low birth rates and increasing longevity pose challenges with regard to the increase of the average employee age along with a lack of skilled personnel on the labor market, organizations are increasingly confronted with the question on how to facilitate successful (i.e., active, healthy, and productive) aging at work, and thus to prolong working lives in the future. Therefore, the aim of the present study was to investigate the relationship between respectful leadership and older workers’ desired retirement age and its underlying mechanisms. In essence, we found that respectful leadership was positively related to older workers’ job satisfaction and subjective health but negatively related to their work-to-private life conflict. Consistent with the recent literature on retirement planning and decision-making (e.g., Fasbender et al., 2014; Pundt et al., 2015; Zhan et al., 2015), this supports the important role of respect and recognition for older people in the workplace.

In addition, the present study highlighted that respectful leadership provides a contextual resource in the retirement decision-making process as it was positively related to desired retirement age. Furthermore, this relationship could be explained by subjective health and work-to-private life conflict. Although the identified relationships are rather small referring to Cohen’s (1992) classification of effect sizes, they can be regarded as quite substantial given the limited variance of desired retirement age (i.e., 55.1% of participants reported their desired retirement age to be 60 years). Job satisfaction, however, although strongly related to respectful leadership, does not seem to play an important role in explaining the relationship of respectful leadership and desired retirement age. The findings add to the literature on the importance of work relationships for older workers, in particular with their leader. In line with SST, older workers are willing to postpone their retirement when experiencing higher levels of subjective health and lower levels of work-to-private life conflict, which are related to the respectful behavior of their leader at work. The desire to retire as opposed to remaining in the workforce reflects the inner process of evaluating one’s resources such as health and potential role conflicts between work and private life and its related socioemotional (resource) gains versus risks reflected by resource threats or losses.

Furthermore, in line with previous research (e.g., Schyns and Collani, 2002; Rigotti et al., 2008), the present study revealed that occupational self-efficacy was positively related to older workers’ job satisfaction and subjective health, and negatively related to their work-to-private life conflict, thus emphasizing the importance of personal resources. However, we found that occupational self-efficacy did not significantly moderate the relationships between respectful leadership and job satisfaction, subjective health, and work-to-private life conflict. Even though we expected that higher levels of occupational self-efficacy would lead people to take an active role in interpreting external factors at work and help them to make better use of existing contextual resources (Lent et al., 1994; Fasbender and Deller, 2017), the present study did not find support for the moderating role of occupational self-efficacy. Future research should continue to investigate occupational self-efficacy and its role in the retirement decision-making process.

### Theoretical and Practical Implications

The findings of the present study extend previous research on retirement decision-making. This study offers relevant theoretical and practical implications. With regard to theory, the present study is among the first to investigate how respectful leadership as a leadership style is linked to older workers’ desired retirement age. While previous studies have already addressed the importance of being treated with respect (e.g., Armstrong-Stassen, 2008), we highlight the role of the leader in the retirement decision-making process as a person with great social influence, who acts according to organizational values of treating older workers with respect and recognition. Thus, respectful leadership as a set of certain behaviors and attitudes (e.g., interest in employees’ opinions, fair treatment, and honest interaction at work) gives employees the feeling of being respected (van Quaquebeke and Eckloff, 2010). Our findings reveal that respectful leadership is a relevant resource for older workers, who, because they perceive time as limited, place greater importance on short-term goals from which they derive socioemotional meaning (SST; Carstensen, 1992, 2006). However, as our sample consisted of older workers, no conclusions can be drawn regarding the relative importance of respectful leadership as a resource for older compared to younger workers. Furthermore, the current findings can inform LMX theory as they show that respect as a central element of high-quality leader–follower relationships is related to retirement intentions. Also, the results support previous findings on the underlying mechanisms in the link between LMX and withdrawal intentions (e.g., Han and Jekel, 2011).

Furthermore, our findings shed light on the role of job satisfaction, subjective health, and work-to-private life conflict in the relationship of respectful leadership and older workers’ desired retirement age. While in our study subjective health and work-to-private life conflict represent relevant resources in this relationship, job satisfaction does not. Although others have found job satisfaction to be related to the intention to remain in the workforce until retirement age (Maurits et al., 2015), in an earlier study Zappalà et al. (2008) also did not find a significant effect of job satisfaction on early versus late retirement intentions. Thus, moderating factors in this relationships as well as effects of measurement should be investigated in future studies. However, in line with SST our findings indicate how important socioemotional gains are for older workers’ retirement decision-making. Future research should continue exploring socioemotional gains and risks or resource gains and losses, respectively, for older workers to understand retirement planning and decision-making in its entirety.

Moreover, our study extends the scarce literature on the interaction of leadership behavior and individual self-efficacy. Although occupational self-efficacy did not moderate the relationship between respectful leadership and several mediators in our study, bivariate correlations have shown that occupational self-efficacy was significantly related to all study variables. Personal resources such as the belief in one’s capabilities therefore seem to be important for the understanding of successful aging at work. Additional research is needed to understand the role of occupational self-efficacy in the relationships between respectful leadership and other organizational factors related to older workers’ desired retirement age. For example, future research could explore the role of autonomy at work as a boundary condition for when occupational self-efficacy facilitates positive work outcomes. It could be assumed that higher levels of autonomy at work enable older workers with high occupational self-efficacy to benefit from respectful leadership behavior in terms of higher job satisfaction, subjective health, but lower work-to-private life conflict, whereas lower levels of autonomy at work may hinder these beneficial effects of occupational self-efficacy. Future research could therefore investigate potential three-way interactions addressing the complex contingency in which retirement decisions are made.

With regard to practice, respectful leadership behavior can help keep older workers in the workforce longer. The study offers insights for potential interventions. Referring to professional development activities, providing trainings for leaders on any level of hierarchy on how to lead their staff respectfully could strengthen this leadership style. Moreover, as older workers elect to engage in activities and relationships at work that facilitate socioemotional meaning to them, they are more likely to benefit from a positive work climate than from new career opportunities or a pay raise. Thus, organizations should facilitate an organizational culture that is driven by respect and recognition toward people of all ages to ensure equally fair treatment for both younger and older workers and avoid potentially arising intergroup conflicts due to positive or negative discrimination. In addition, organizations can offer professional development activities to their employees. An intervention study has shown that through coaching employees’ work ability could be improved (McGonagle et al., 2014). Although the authors failed to find a significant improvement in occupational self-efficacy, it points to the trainable nature of beliefs regarding own capabilities. For example, strengthening the older workers’ occupational self-efficacy through work specific training, such as allowing workers to sense achievements in their job, may help reinforce their subjective health and motivational outcomes. Together, these interventions may support the voluntary extension of working lives.

### Limitations and Directions for Future Research

Notwithstanding the theoretical contribution of our findings, we acknowledge some limitations of this research and refer to directions for future research. First, the cross-sectional nature of the data does not allow drawing causal inferences. It is possible that the relationship between respectful leadership and desired retirement age is reversed as other influences, such as socioeconomic factors (e.g., financial dependency) may determine older workers’ desired retirement age, which, in turn, could lead them to evaluate their leaders more favorably (i.e., to reduce cognitive dissonance; Festinger, 1957). Therefore, future research should use longitudinal data to allow for a more precise inference about causality and/or reciprocal relationships, in particular with regard to the understanding of older workers’ retirement planning and decision-making.

Second, the current study relies on self-report measures which are thought to have shortcomings (Podsakoff et al., 2003). However, older workers’ indication of their desired retirement age was measured as open text response (not via scale). In this case, it is rather unlikely that self-report produces inaccurate or systematically biased answers, which partly alleviates the concern for common-method bias (Spector, 1994; Zhan et al., 2015; Fasbender et al., 2016). Furthermore, procedural means were used to control for common method variance (Zaniboni, 2015); ensuring participants’ anonymity was protected with regard to their employer. Participants were recommended to answer questions honestly, and they were advised that there were no right or wrong answers. Moreover, a moderation variable was considered to increase complexity, diminishing the threat of participants’ “theory-in-use” (Chang et al., 2010). Nevertheless, future research should additionally consider data from other sources than older workers’ themselves, for example, by involving the perspective from leaders on their behaviors, attitudes, and leadership style. Furthermore, in this study we focused on the desired retirement age as part of the retirement decision-making process. Including the actual retirement age in future research would enhance the knowledge on the impact of the investigated processes on actual behavior.

The present study also leaves some issues unaddressed, suggesting areas for further investigation. Because this study was tailored toward the legal and economic environment of Germany, future research should replicate our findings in other countries. In particular, it is relevant to understand whether the desired retirement age may differ across cultures due to varying insurance and pension systems. In addition, research could address how respectful leadership is related to organizational culture and individuals’ work ethics, and how this relates to retirement decision-making. Also, it would be illuminating to explore how different personality traits interact with this leadership style, and how this, in turn, may impact the desire to retire.

## Ethics Statement

This study was carried out in accordance with the ethical guidelines of the Leuphana University, Lüneburg with an informed consent from all study participants. Full review and approval of the study was not required according to the local and national regulations and guidelines.

## Author Contributions

AW did the research design, data collection, data analyzing, theorizing, and writing. UF did the data analyzing, theorizing, and writing. JD did the research designing, data collecting, and critical revising.

## Conflict of Interest Statement

The authors declare that the research was conducted in the absence of any commercial or financial relationships that could be construed as a potential conflict of interest.
